# An Extended Approach to Quantify Triacylglycerol in Microalgae by Characteristic Fatty Acids

**DOI:** 10.3389/fpls.2017.01949

**Published:** 2017-11-13

**Authors:** Miao Yang, Yan Fan, Pei-Chun Wu, Ya-Dong Chu, Pei-Li Shen, Song Xue, Zhan-You Chi

**Affiliations:** ^1^School of Life Sciences and Biotechnology, Dalian University of Technology, Dalian, China; ^2^Marine Bioengineering Group, Dalian Institute of Chemical Physics, Chinese Academy of Sciences, Dalian, China; ^3^University of Chinese Academy of Sciences, Beijing, China; ^4^State Key Laboratory of Bioactive Seaweed Substances, Qingdao Bright Moon Seaweed Group Co., Ltd., Qingdao, China

**Keywords:** microalgae, triacylglycerol, quantification, characteristic fatty acid, linear correlation

## Abstract

Microalgae represent a third generation biofuel feedstock due to their high triacylglycerol (TAG) content under adverse environmental conditions. Microalgal TAG resides in a single cell and serves as a lipid class mixed with complicated compositions. We previously showed that TAG possessed characteristic fatty acids (CFAs) for quantification and was linearly correlated with the relative abundance of CFA within certain limits in microalgae. Here, we defined the application range of the linear correlation between TAG and CFA in the oleaginous microalgae *Chlamydomonas reinhardtii* and *Phaeodactylum tricornutum*. In addition, TAG quantification was further expanded to a wide range of levels and the absolute amounts of saturated or monounsaturated CFAs, 16:0 and 18:1n9 of *C. reinhardtii* and 16:0 and 16:1n7 of *P. tricornutum*, instead of polyunsaturated CFAs, were verified to be linearly correlated to TAG levels throughout the entire period of nitrogen stress. This approach utilizes a single fatty acid to quantify TAG mixtures, and is rapid, simple and precise, which provides a useful tool for monitoring TAG accumulation of distinct microalgal species and facilitating high-throughput mutant screening for microalgae.

## Introduction

Microalgae have emerged as a promising renewable resource for producing triacylglycerols (TAGs), which can be used as biofuel feedstocks (Wijffels and Barbosa, 2010). Nitrogen starvation is the most effective manner to induce microalgal TAG biosynthesis (Zienkiewicz et al., 2016). It is necessary to investigate performance of TAG accumulation under distinct levels of nitrogen stress in microalgae to screen potential candidates for oleaginous species. However, microalgae are different from other oleaginous feedstocks, such as plant seeds, of which the lipids are dominated by TAG. In particular, microalgae exist in form of a single cell and contain various lipids with different polarities. In most microalgal cells, the chloroplast serves as the largest organelle and contains many complicated and hydrophobic components, which makes TAG quantification more intricate. Therefore, it is desirable to develop an approach to efficiently quantify TAG content of microalgal biomass under various growth conditions.

There are several methods to determine the TAG content of microalgal biomass. The gravimetric method of Bligh and Dyer (1959) is widely used to evaluate the lipid content in microalgae. Using this method, many other liposoluble components, including pigments and other hydrophobic compounds, can be co-extracted. With the aid of solid phase extraction (SPE) column, neutral lipids that are mainly composed of TAG can be separated from polar lipids (Li et al., 2010; Danielewicz et al., 2011). However, the two extraction methods are both dependent on the similarity-intermiscibility theory and lead to overestimation of microalgal TAG levels (Na et al., 2011). Another commonly used approach is based on thin layer chromatography (TLC) separation followed by gas chromatography-flame ionization detection (GC-FID) or a TLC scanner in terms of densitometry quantification (Siaut et al., 2011), which is time and labor consuming or requires specialized expensive equipment, as well as not applicable for assessing TAG phenotypes in real-time. TAG can also be quantified by high performance liquid chromatography (HPLC) (Kobayashi et al., 2013), direct mass spectrometry (MS) (Danielewicz et al., 2011) and liquid chromatography-mass spectrometry (LC-MS) (Goold et al., 2016), but these methods are complicated due to the requirement of a lipid extraction pre-treatment and their complex molecule compositions accompanied with intricate data treatment. A specific quantification method (Allen et al., 2014) is also developed for algal TAG using LC-MS/MS in multiple reaction monitor mode and the incorporation of catalytic hydrogenation reduces complexity of targeted TAG pool; however, the relevant pre-treatment together with relatively complex data analysis are still of multiple steps, to a certain extent. Raman microspectroscopy (Wang T. et al., 2014), spontaneous Raman spectroscopy and coherent anti-Stokes Raman scattering (CARS) microscopy (He et al., 2012), Fourier transform infrared (FT-IR) spectroscopy (Miglio et al., 2013) and thermogravimetric analyses (Na et al., 2011) have also been used to monitor TAG production. Although there is no need to extract lipids from microalgal biomass using these methods, non-TAG molecules with similar biochemical bonds or thermal features can easily cause interference, which leads to inaccurate quantification.

To date, it has been reported that microalgal oils can be determined by direct transesterification of fresh or dry algal samples (Patil et al., 2013; Liu et al., 2015), which is rapid and precise, and has minor sample demands. Based on this technique, the specific fatty acids, 18:1 and 18:4, were identified as potential biomarkers of neutral lipids in *Isochrysis zhangjiangensis* and linear fits existed between the relative abundances of the specific fatty acids and the neutral lipid content as reported by our previous study (Wang H. et al., 2014). As the major components of neutral lipids, TAGs are mainly comprised of saturated or monounsaturated fatty acyl groups (Siaut et al., 2011), which are considered as the potential feedstocks for biodiesel production. Thus, quantifying TAG is indispensable for microalgal studies in terms of their use as renewable resources. Our recent study further showed that TAG could be quantified using the relative abundance of characteristic fatty acids (CFA) and an excellent linear correlation existed between them, which has been verified in three microalgal strains, including *Phaeodactylum tricornutum*, *Nannochloropsis oceanica* and *Chlorella pyrenoidosa* (Shen et al., 2016). Additionally, Liu et al. (2013) reported that, in *Chlamydomonas reinhardtii*, the 16:0-to-16:4 ratio in fatty acid methyl esters (FAMEs) was strongly correlated with the TAG-to-total acyl group ratio, which serves as an internal standard for TAG estimation. However, TAG quantification using fatty acids in these studies are only available within certain limits, usually when TAG accumulation is at a moderate or relatively low level, and the application range still remains unknown.

Based on these studies, the TAG has been validated to possess CFAs for quantification in microalgae. Many algal strains, including oleaginous and non-oleaginous species, present significant alterations in the fatty acyl profile in response to nitrogen deprivation along with an increase of TAG level (Breuer et al., 2012). This stage for TAG assembly is named Stage I. However, after that, the fatty acid profile is unaltered and TAG continues to accumulate (Breuer et al., 2012). At this point, the quantitative approach for TAG based on the relative abundance of CFA in our previous work (Shen et al., 2016) is no more available, and this period for TAG synthesis is named Stage II. Thus, it is crucial to evaluate TAG accumulation performance using CFAs in an expanding coverage, so that the TAG levels of microagal cells during distinct stages (Stage I and II) of TAG accumulation can be rapidly and accurately quantified.

Currently, *C. reinhardtii* has emerged as the leading organism to investigate algae-based biofuel production due to its most available and multiple genetic tools and techniques (Merchant et al., 2007; Scranton et al., 2015). Moreover, starchless mutants deficient in ADP-glucose pyrophosphorylase harbor a unique feature of hyper-accumulating TAG (Li et al., 2010). The model marine diatom *P. tricornutum* serves as a potential producer for biodiesel due to its high TAG accumulation capability and robust environmental adaptation (Breuer et al., 2012). The available genetic tools and genome engineering techniques have empowered this oleaginous microalgal strain for industrial biofuel production (Daboussi et al., 2014).

In this study, the two model microalgal strains *C. reinhardtii* and *P. tricornutum* were used to correlate the TAG content with the fatty acyl composition under distinct levels of nitrogen stress. During the entire period of nitrogen starvation, different relationships between the TAG levels and fatty acid components were explored, and the respective CFAs were confirmed to rapidly quantify the TAG amounts in these two algae. This extended approach to quantify distinct levels of TAG using CFAs was successfully applied to these two reference species and could serve as a useful tool for monitoring TAG accumulation of multiple microalgal species and facilitating high-throughput mutant screening for microalgae.

## Materials and Methods

### Microalgal Cultivation

The *C. reinhardtii* cell wall-less strain, starchless mutant BAFJ5 (cw15 sta6, CC4348) was obtained from the Chlamydomonas Resource Center^1^. The *P. tricornutum* strain CCAP1055/1 was provided by the Culture Collection of Algae and Protozoa^2^. These two strains were maintained in a 500-mL Erlenmeyer flask with 200 mL of Tris-acetate-phosphate (TAP) medium (Harris, 2009) under orbital shaking (80 rpm) and in a 1000-mL Erlenmeyer flask with 400 mL of f/2 medium containing 1 M sodium silicate (Guillard, 1975), respectively. They were both sub-cultured under a 12 h light/12 h dark cycle at 25°C with an illumination of 50 μmol photons m^-2^ s^-1^, and the illumination intensity was determined by a photosynthetically active radiation (PAR) detector (Optometer P9710, Gigahertz Optik Corporation, Germany).

Two-stage cultivations, including the first nitrogen repletion phase and the subsequent nitrogen-deprived phase, were used to induce TAG accumulation. Illumination was set as 50 μmol photons m^-2^ s^-1^ during N-replete cultivation. *C. reinhardtii* was cultured in TAP medium for 48 h under continuous illumination and *P. tricornutum* was cultured in 3 × f/2 medium containing 3 M sodium silicate (Feng et al., 2011) for 48 h under a 12 h light/12 h dark cycle. The batch culture mode was performed in a glass air bubble column photobioreactor (50 mm diameter, 450 mm height, 600 mL for a culture volume) with air (120 mL min^-1^) containing 2% CO_2_. When two algal cell populations grew to 1–2 × 10^7^ cells mL^-1^ under N-replete conditions, *C. reinhardtii* and *P. tricornutum* were washed once with TAP-N and 3 × f/2-N medium, respectively, and inoculated at 1 × 10^7^ cells mL^-1^ in the corresponding medium. During N-deprived cultivation for *C. reinhardtii* and *P. tricornutum*, the illuminations provided from one side rose to 100 and 500 μmol photons m^-2^ s^-1^ to enhance TAG accumulation, respectively, and the light cycles were the same as that used under the N-replete conditions.

One independent batch culture that included two biological replicates for *C. reinhardtii* and *P. tricornutum* was conducted. *C. reinhardtii* was sampled at a total of 30 time points over 120 h of N-deficiency. *P. tricornutum* was sampled at 21 time points during the light period over 336 h of N-starvation. The samples were first centrifuged at 4000 rpm for 5 min, and the pellets were immediately frozen at -80°C, followed by lyophilization for 4 h. In particular, pellets of *P. tricornutum* were rinsed with ammonium bicarbonate (0.5 M) to remove extracellular salt before lyophilization. After grinding cells into powder, the algal biomass was stored at -80°C for subsequent lipid and element analysis.

### Lipid Analysis

The lyophilized algal powders were used to detect the fatty acid profiles or contents, and TAG contents. For the fatty acid profiles, algal biomass was converted to FAMEs through direct transesterification, followed by GC determination as previously reported (Liu et al., 2015). Briefly, approximately 5 mg of lyophilized cells were weighed using an analytical balance (MSE125P-1CE-DI, Sartorius, Germany). Five milliliters of 2% H_2_SO_4_ in methanol were added, and the mixture was heated at 70°C for 1 h. FAMEs were extracted by hexane and quantified using an Agilent GC 7890A fitted with FID and a DB-23 column (Agilent Technologies, United States). Glycerol triheptadecanoate (TAG 51:0, 17:0/17:0/17:0, Sigma–Aldrich, United States) was used as an internal standard to determine fatty acid recovery for quantification.

Unlike detection of fatty acid profiles or contents, TAG quantification required additional two steps beyond transesterification, including total lipid extraction from biomass (Bligh and Dyer, 1959) and lipid separation by TLC. Methanol:chloroform:water (1:1:0.9) was used as the extraction solvent. A 950 μL extraction solvent composed of methanol:chloroform:water (1:2:0.8) was first added to pre-weighed lyophilized cells with a pre-addition of the lipid standard TAG 51:0 followed by a 15-min sonication. After a complete mixing, an additional addition of 250 μL chloroform was mixed into the solvent, followed by a subsequent addition of 250 μL H_2_O. The samples were vortexed and centrifuged for 2 min at 12,000 rpm. The organic phase of the lower layer was then transferred to a 2 mL glass vial. The lipid extraction process was repeated two more times and the extracted lipids were pooled and dried under a gentle stream of N_2_. Next, 100 μL chloroform were added to redissolve the total lipids, and the lipid extracts were deposited onto a TLC plate (10 cm × 10 cm, TLC silica gel 60 F254, Merck KGA, Germany). The TLC plate was developed with hexane/diethyl ether/acetic acid (85:15:1, v/v/v), and the lipids were revealed by spraying with 0.05% (m/v) primuline (Sigma–Aldrich, United States) in acetone/water (80/20, v/v). The silica-containing TAG was scrapped off, followed by transesterification and GC detection.

The individual fatty acid percentage of the cellular total acyl groups and, the individual fatty acid and TAG contents based on the cellular dry weight (DW) were calculated as follows:

$$P_{i}=A_{i}/{\Sigma A}_{i}\times 100\%$$

$$M_{i}=\left(A_{i}/A_{s}\times M_{s}\right)/M_{a}\times 100\%$$

$$M_{TAG}=\left(\Sigma A_{i}/A_{s}\times M_{s}\right)/M_{a}\times 100\%$$

where P_i_, M_i_ and M_TAG_ were the individual fatty acid percentage of total fatty acids, fatty acid content and TAG content, respectively. A_i_ and ΣA_i_ in Equation 1 were the peak area of the individual fatty acid and that of cellular total fatty acids, respectively. ΣA_i_ in Equation 3 was the peak area of total fatty acyls of TAG. A_s_, M_s_ and M_a_ in Equations 2 and 3 were the peak area of the internal standard TAG 51:0, the addition amount of TAG 51:0 and the accurate algal biomass, respectively.

### Elemental Analysis

Quantification of carbon and nitrogen of the algal biomass was performed using a vario EL cube elemental analyzer (Elementar, Germany). Two to four milligrams of lyophilized algal cells were accurately weighed using an automatic analytical balance (Mettler Toledo XP6, Switzerland).

### Statistical Analysis

Correlation coefficients between CFAs and TAG contents were calculated using SPSS 19.0. Statistical significance of the result of each time point was evaluated by the Two-tailed *t*-test using SPSS 19.0, where the significance *P* < 0.05.

## Results

### Variation of Fatty Acid Profiles and TAG Contents of *C. reinhardtii* and *P. tricornutum* under Distinct Levels of Nitrogen Stress

**FIGURE 1:**
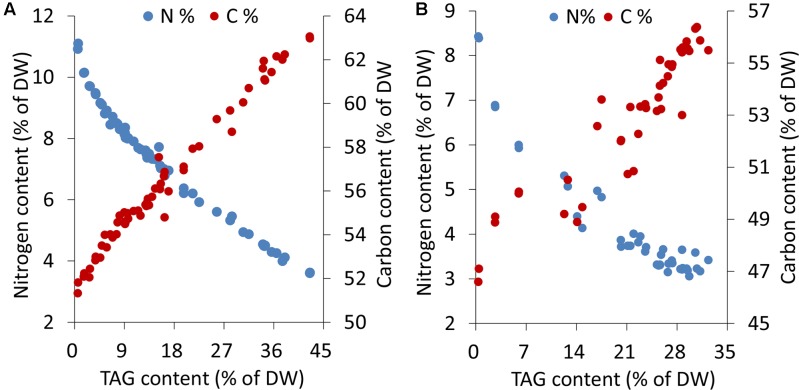
Elemental analyses and TAG contents in *C. reinhardtii* **(A)** and *P. tricornutum* **(B)** under nitrogen deprivation. The sampling time points were 30 for *C. reinhardtii* **(A)** and 21 for *P. tricornutum* **(B)**. Each time point was derived from two biological replicates of a single cultivation, and each data point was drawn from an individual result rather than the mean of the two biological replicates (*n* = 60, **A**; *n* = 42, **B**).

Microalgal cells underwent different levels of stress during an extended period of nitrogen deprivation. Elemental analyses indicated that the cellular nitrogen contents decreased by 68%, from 11.1 to 3.6% of DW, in *C. reinhardtii* and 64%, from 8.4 to 3.0% of DW, in *P. tricornutum*. The carbon contents of both *C. reinhardtii* and *P. tricornutum* increased by 1.2-fold following N-starvation (**Figure 1**). In both algae, as the stress levels gradually rose, a sustaining accumulation of TAG occurred.

**FIGURE 2:**
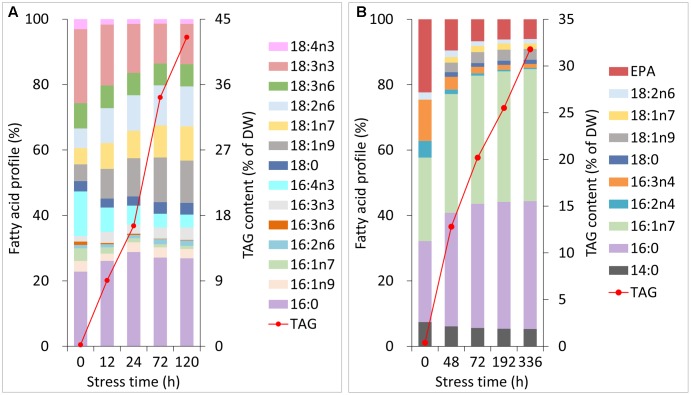
Changes in the fatty acid profiles and TAG contents of *C. reinhardtii* **(A)** and *P. tricornutum* **(B)** under N-deprived conditions. Data are means of two biological replicates with two technical replicates (*n* = 4) from one independent cultivation.

During normal growth of *C. reinhardtii* and *P. tricornutum*, the primary fatty acyl groups of the former consisted of 16:0, 16:4n3 and 18:3n3, and those of the latter consisted of 16:0, 16:1n7, 16:3n4 and 20:5n3 (eicosapentaenoic acid, EPA). Each of the fatty acids accounted for more than 10% of the total lipids (**Figures 2A,B**). During Stage I of TAG accumulation (less than 24 h for *C. reinhardtii* and 72 h for *P. tricornutum* in this study), the fatty acid compositions of the two algal cells varied notably as the nitrogen stress levels grew (**Figures 2A,B**). At the end of Stage I, the relative abundances of 16:0, 18:1n9, 18:1n7 and 18:2n6 increased by 1.3-, 2.3-, 1.7- and 1.8-fold in *C. reinhardtii*, respectively. Inversely, 16:4n3 and 18:3n3 decreased to 40 and 70% of the initial levels, respectively. In *P. tricornutum*, both 16:0 and 16:1n7 rose up to 1.6-fold of the initial levels and 16:3n4 and EPA sharply declined to 20 and 30% after 72 h of N-depletion, respectively. At this point, these two microalgae accumulated TAGs up to 17% for *C. reinhardtii* and 20% for *P. tricornutum* based on DW (**Figures 2A,B**). During Stage II (longer than 24 h for *C. reinhardtii* and 72 h for *P. tricornutum* in this study), the relative fatty acid percentages of these two algae were almost unaltered; however, TAG substantially accumulated up to 43% for *C. reinhardtii* and 32% for *P. tricornutum* based on DW (**Figures 2A,B**).

### Quantification of TAG Using the Relative Abundance of CFA in *C. reinhardtii* and *P. tricornutum*

**FIGURE 3:**
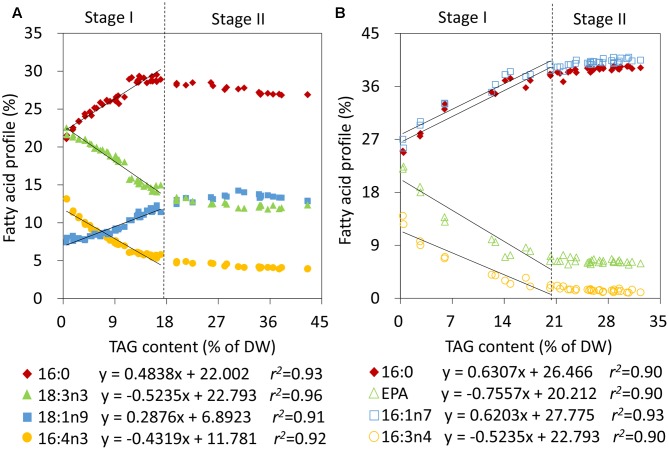
Correlation analyses of the relative abundances of fatty acids and TAG contents in *C. reinhardtii* **(A)** and *P. tricornutum* **(B)** under nitrogen deprivation. The sampling time points were 30 for *C. reinhardtii* **(A)** and 21 for *P. tricornutum* **(B)**. Solid lines indicated the linear fits between TAG contents and the relative abundances of CFAs at Stage I and dashed lines indicated the boundary of Stage I and Stage II. In the linear equations, the variable “x” is the TAG content, “y” is the relative fatty acid percentage, and “*r^2^*” is the correlation coefficient of x and y. The linear equations were derived from individual results of 19 time points (*n* = 38) within 24 h of nitrogen starvation for *C. reinhardtii* and 7 time points (*n* = 14) within 72 h of nitrogen starvation for *P. tricornutum*. Each time point was derived from two biological replicates of a single cultivation, and each data point was drawn from an individual result rather than the mean of the two biological replicates (*n* = 60, **A**; *n* = 42, **B**).

To obtain quantitative models of TAG during the two stages, the relative abundances of fatty acids and TAG levels were first correlated in *C. reinhardtii* and *P. tricornutum* that were subjected to nitrogen stress. During Stage I, linear fits were observed between the relative percentages of 16:0, 16:4n3, 18:1n9 as well as 18:3n3 and the TAG contents in *C. reinhardtii*, and the relevance coefficients *r^2^* were 0.93, 0.92, 0.91, and 0.96, respectively (**Figure 3A**). 16:0 and 18:1n9 were found to be positively correlated with the amounts of TAG and 16:4n3 and 18:3n3 were on the contrary (**Figure 3A**). During stage II, the TAG level exceeded 17% of DW and the proportions of the four fatty acids of total lipids were maintained at 28 ± 1%, 4 ± 0%, 13 ± 1% and 13 ± 1%, respectively (**Figure 3A**). Obviously, the above linear models were no longer applicable. Similarly, in *P. tricornutum*, four fatty acids, 16:0, 16:1n7, 16:3n4 and EPA, were shown to be linearly related to the TAG contents (**Figure 3B**) and the *r^2^* values were all more than 0.90 during Stage I. A positive relationship existed between 16:0 as well as 16:1n7, and TAG, and there was a negative correlation between 16:3n4 as well as EPA, and TAG in *P. tricornutum* (**Figure 3B**). Beyond these limitations, the four fatty acyl groups were stable at 39 ± 1%, 40 ± 1%, 1 ± 0% and 6 ± 0%, respectively (**Figure 3B**). These linear correlations indicated that 16:0, 16:4n3, 18:1n9 as well as 18:3n3 of *C. reinhardtii*, and 16:0, 16:1n7, 16:3n4 as well as EPA of *P. tricornutum* were CFAs to quantify TAG, but it was only limited to Stage I of nitrogen stress in both two algae.

### Quantification of TAG Using the Absolute Amount of CFA in *C. reinhardtii* and *P. tricornutum*

**FIGURE 4:**
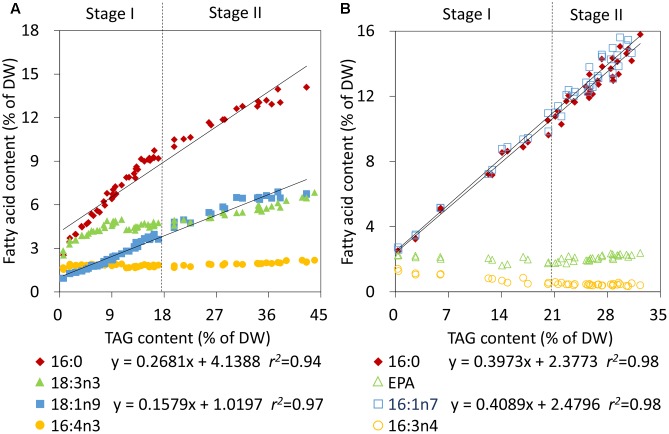
Correlation analyses of the fatty acid contents and TAG contents in *C. reinhardtii* **(A)** and *P. tricornutum* **(B)** under nitrogen deprivation. The sampling time points were 30 for *C. reinhardtii* **(A)** and 21 for *P. tricornutum* **(B)**. Solid lines indicated the linear fits between TAG contents and the absolute amounts of CFAs during the entire phase of nitrogen starvation, including Stage I and Stage II, and dashed lines indicated the boundary of Stage I and Stage II. In the linear equations, the variable “x” is the TAG content, “y” is the CFA content, and “*r^2^*” is the correlation coefficient of x and y. The linear equations were derived from individual results of 30 time points (*n* = 60) for *C. reinhardtii* and 21 time points (*n* = 42) for *P. tricornutum*. Each time point was derived from two biological replicates of a single cultivation, and each data point was drawn from an individual result rather than the mean of the two biological replicates (*n* = 60, **A**; *n* = 42, **B**).

To further rapidly quantify TAG also using CFA during Stage II in nitrogen-deprived *C. reinhardtii* and *P. tricornutum*, the individual CFA content based on DW was correlated with the TAG content. The individual CFA identified for Stage I was quantified in terms of the internal standard and the accurate microalgal biomass, which was calculated according to Equation 2 (see Materials and Methods). The absolute amounts of 16:0 and 18:1n9 of *C. reinhardtii* and 16:0 and 16:1n7 of *P. tricornutum* all showed linear fits with the increased TAG levels during both Stage I and II (**Figures 4A,B**). The relevance coefficients of 16:0 and 18:1n9 with TAG for *C. reinhardtii* were 0.94 and 0.97 (**Figure 4A**), and the coefficients for 16:0 and 16:1n7 with TAG for *P. tricornutum* were both 0.98 (**Figure 4B**). However, by contrast, the polyunsaturated CFAs of Stage I, including 16:4n3 and 18:3n3 of *C. reinhardtii* as well as 16:3n4 and EPA of *P. tricornutum*, exhibited no such fits with TAG. Therefore, using the linear equations in **Figure 4**, the TAG contents (x) could only be determined quickly and precisely by the continuously increasing saturated or monounsaturated CFAs (y).

## Discussion

### Linear Correlation between TAG and CFA during Distinct Stages of Nitrogen Stress in Microalgae

In microalgae, the neutral lipid, TAG, has been verified to possess CFA for quantification and the relative abundance of CFA can be used to linearly quantify TAG within certain limits under stress conditions (Shen et al., 2016). However, TAG consecutively accumulates as stress level increases, and it is inconsistent with the variation of fatty acid profile, i.e., gradual alteration followed by invariability, in stress-induced microalgae (Breuer et al., 2012). Thus, it is necessary to define applicability and expand coverage of the CFA-based TAG quantification method established in our previous work (Shen et al., 2016). To expand its application range, the correlations between TAG and CFA were further explored over the entire period of nitrogen stress in two oleaginous microalgae *C. reinhardtii* and *P. tricornutum*.

Under stress condition, microalgae usually encompasses gradual chloroplast degradation and remodeling of the lipidome, accompanied by substantial accumulation of TAG (Zienkiewicz et al., 2016); all these alterations affect the fatty acid compositions of microalgae prominently. In this study, the fatty acid profile of algal biomass changed gradually and then remained constant upon nitrogen starvation, i.e., Stage I and Stage II, which agreed with the previous report (Breuer et al., 2012). Thus, TAG accumulation underwent two stages in terms of the fatty acid profile and the constant fatty acid profile became the determinant to distinguish Stage I and Stage II of nitrogen stress. At the turning point, the TAG levels of *C. reinhardtii* and *P. tricornutum* reached up to 17 and 20% of DW, respectively (**Figure 3**). The results in this study confirmed that the TAG quantification method based on the relative abundance of CFA (Shen et al., 2016) was only applied to Stage I. More importantly, it was the absolute amount of saturated or monounsaturated CFA, e.g., 16:0 and 18:1n9 of *C. reinhardtii* and 16:0 and 16:1n7 of *P. tricornutum*, instead of the relative abundance, to be linearly correlated to TAG content over the entire period of nitrogen deprivation. In addition, the polyunsaturated CFA could be used to quantify TAG only at Stage I and only when the relative abundance was available.

In this study, the proportions of TAG in total lipids exhibited gradual increases from 6 to 81% for *C. reinhardtii* and from 4 to 83% for *P. tricornutum* in response to nitrogen starvation (**Supplementary Figure S1**). In these two microalgae, TAGs were mainly composed of saturated and monounsaturated fatty acyls (Supplementary Tables S1, S2), which were presumed to be *de novo* synthesized (Liu et al., 2013). These newly synthesized fatty acids were mostly diverted into TAG following N-depletion. Thus, the concurrent increases of the absolute amounts of 16:0 and 18:1n9 of *C. reinhardtii* and 16:0 and 16:1n7 of *P. tricornutum* as well as the respective TAG contents based on dry biomass led to the formation of the linear correlations during the entire stage of nitrogen stress. It also demonstrated that the quantitative saturated or monounsaturated CFA was the authentic CFA, which could be utilized to quantify TAG at any levels.

Apart from a slight increase of 18:3n3 of *C. reinhardtii*, the contents of all of the polyunsaturated CFAs, 16:4n3 of *C. reinhardtii* and 16:3n4 and EPA of *P. tricornutum*, based on DW were almost invariable (**Figure 4**), which agreed well with previously reported results (Boyle et al., 2012; Remmers et al., 2017). These polyunsaturated CFAs were primarily located in photosynthetic membranes and played crucial roles in stabilizing photosystems (Petroutsos et al., 2014). A portion of polyunsaturated fatty acids was transferred from polar lipids to TAG when subjected to adverse conditions (Remmers et al., 2017), which could protect them from peroxidation. These factors contributed to stabilizing the amount of polyunsaturated fatty acids following nitrogen stress, yet they were no longer qualified to be CFAs for TAG quantification during Stage II.

Normally, *de novo* fatty acid synthesis together with various membrane lipids turnover contributes to TAG biosynthesis (Goold et al., 2016). In this study, no obvious alterations in acyl compositions of the major lipids, MGDG, DGDG and DGTS, were found during Stage I and Stage II of TAG accumulation in nitrogen-deprived *C. reinhardtii* as a whole (**Supplementary Figure S2**). However, in contrast to the greatly increased TAG, the levels of the three major lipids varied slightly (**Supplementary Figure S3**) in stress-induced *C. reinhardtii*. At the turning point of TAG accumulation (24 h of nitrogen deprivation), TAG levels of *C. reinhardtii* were almost equal to the polar lipid levels. During Stage I, TAG accumulation appeared to be intimately correlated with contribution of various polar lipids, especially the newly synthesized DGDG and DGTS (**Supplementary Figure S3**). It is likely that more than one pathway involving distinct membrane lipids differentially participated in TAG assembly during Stage I, which resulted in a notable variation in the lipid profile. As TAG level was further elevated, TAG accumulation entered into Stage II and the fatty acid profile was no longer altered. During Stage II, TAG dramatically accumulated up to the level that was much more than that of polar lipids, and thus TAG formation was mainly attributed to the *de novo* biosynthesis, instead of turnover of membrane lipids. These findings suggested that the TAG biosynthesis pathways reached equilibrium. Therefore, Stage II was a period of lipid homeostasis for microalgae, which implied a balanced status of multiple TAG biosynthetic pathways. Overall, the turnover of membrane lipids together with the *de novo* biosynthesis pathway differentially contributes to TAG biosynthesis during different stages of TAG accumulation in microalgae. This could help understand the origin of CFAs and the distinct TAG biosynthesis mechanisms, which provides insights into their linear correlation with TAG content in the algal cell.

### Principle of the CFA-Based Method to Quantify TAG in Microalgae

The current study validated two sets of correlations between CFA and TAG levels, and the correlations were applied to two stages of TAG accumulation for two model microalgae *C. reinhardtii* and *P. tricornutum*. First, it was necessary to identify the TAG accumulation stage based on the microalgal fatty acid profiles, which just required direct transesterification. Or, it could be judged in terms of the known stress time, i.e., short or long-term stress. Subsequently, the appropriate method for TAG quantification was confirmed to rapidly evaluate TAG accumulation in microalgae. If the fatty acid profile was coincided with that of Stage I, there was just a need to calculate TAG content using linear equation of that and the relative abundance of any CFA in **Figure 3**. If the fatty acid profile matched with that of Stage II, it was required to perform the transesterification process again and additional operations, including the accurate additions of fresh or dry algal biomass and internal standard of known weight, were necessary to calculate TAG content using linear equation of that and the absolute amount of any CFA in **Figure 4**. Alternatively, the absolute amount of the saturated or monounsaturated CFA, i.e., 16:0 and 18:1n9 of *C. reinhardtii* and 16:0 and 16:1n7 of *P. tricornutum*, can always be used to quantify TAG during both Stage I and Stage II of nitrogen stress. In this case, the relative abundance of CFA can be neglected, but the accurate additions of algal biomass and internal standard of known weight is necessary for each determination.

No matter which linear equation is used, TAG content can be rapidly determined without either prior lipid extraction or succeeding lipid separation using SPE column or TLC plate and it only needs one-step transesterification and GC-FID analysis, which is in favor of the algal lipid researchers to monitor TAG accumulation in real-time. This approach requires less than 1.5 h for one determination. If one wishes to track the TAG contents over a period of time, it is feasible to perform direct transesterification using fresh cells and GC-FID analysis in the meantime. Additionally, the algal samples for TAG quantification can be fresh or dry cells and a minimum of biomass equivalent to 30 μg lipids can meet demands as reported by our previous study (Liu et al., 2015), which is much less than the classical TLC versus GC-FID method. As to the emerging LC-MS method, TAG is a mixture of various components and it is both time-consuming and expensive to separate and detect many molecular species of TAG using HPLC and MS; the accurate quantification of the mixture also needs complicated data treatment even a quality control correction in terms of TLC-GC analysis (Jouhet et al., 2017). In comparison, direct transesterification coupled with GC detection of certain fatty acids is quicker and simpler. Overall, this extended CFA approach utilizes a single fatty acid to quantify TAG mixtures and can facilitate high-throughput mutant screening for algae to a certain extent, which not only simplifies the process but also ensures its accuracy.

It is worth noting that the fatty acyl profiles of microalgae from distinct labs are probably different, and thus, the TAG amounts quantified using CFAs might be somewhat different due to distinct culture conditions and microalgal species specificities. Therefore, it is preferable to establish the corresponding quantitative relationships between CFA and TAG for the targeted strains cultured under the designated conditions, which is convenient for researchers to quickly assess the TAG levels and facilitate relevant biochemical or physiological studies.

As a whole, the present work is to establish distinct linear equations of TAG and CFA across distinct stages of TAG accumulation with emphasis on extensive application, though our previous work is to identify CFAs for TAG quantification and confirm correlation between CFA and TAG content with emphasis on the concept of “CFA.” This study not only expands coverage of TAG quantification based on distinct linear equations, but also applies to an alga mutant that enhances oil biosynthesis, i.e., *sta6* of a starchless *C. reinhardtii* mutant, which both increase impact of the CFA approach. The successful application of this method on the mutant shows its usefulness for the potential forward genetics study, which could also be expanded to other mutant algal species.

To further increase impact of the CFA approach for TAG quantification, we checked the correlations of TAG and CFA under phosphate deprivation for *Nannochloropsis oceanica* and high salt stress conditions (HS medium, photoautotraphic conditions) for *sta6* of *C. reinhardtii*. The results revealed that the excellent linear relationships also existed between TAG and CFA and the linear correlation coefficients were all more than 0.90 under both the two stress conditions (**Supplementary Figures S4**, **S5**), which further confirmed validity and wide use of the CFA method. While these linear correlations were consistent with that under nitrogen stress, they were not completely the same due to the fact that TAG accumulation mechanisms differed among various stress conditions. Beyond that, the linear correlations of TAG contents and the absolute amounts of CFAs (16:0 and 18:1) were also verified in the reported studies of Lohman et al. (2014) and Wase et al. (2017). Thus, the CFA method works not only for the nitrogen induced TAG production, but also for other stress conditions, e.g., high salt, phosphate starvation, different inorganic carbon regimes (Lohman et al., 2014) and even the various chemical activators (Wase et al., 2017). Based on our present study and the published data, the linear correlation of TAG and CFA is proved to be available for more than one stress condition, although the corresponding linear equation for TAG quantification is case by case under distinct stress conditions.

## Conclusion

An extended approach to quantify distinct levels of TAG using CFA was successfully applied to two oleaginous microalgae, *C. reinhardtii* and *P. tricornutum*. It not only defines the application range of the linear correlation between TAG and the relative abundance of CFA, but also expands coverage of TAG quantification in microalgae. The absolute amount of saturated or monounsaturated CFA is verified to be linearly correlated with TAG content over the entire period of nitrogen stress. This approach utilizes a single fatty acid to quantify TAG mixtures, and is rapid, simple and precise. Moreover, it can be widely applied to various microalgal species with variable TAG levels, monitor TAG accumulation in real-time and facilitate high-throughput mutant screening for microalgae.

## Author Contributions

MY and SX conceived and designed the research. MY performed the experiments and analyzed data. MY and SX wrote the manuscript. YF and P-CW participated in algal cultivations. Y-DC designed the photobioreactors and illumination systems. P-LS provided technical assistance. SX and Z-YC supervised specific experiments. All authors agreed on the manuscript.

## Conflict of Interest Statement

The authors declare that the research was conducted in the absence of any commercial or financial relationships that could be construed as a potential conflict of interest.

## Footnotes

1 http://www.chlamycollection.org/

2 https://www.ccap.ac.uk/

## Supplementary Material

**FIGURE S1 |** Time course alterations of the fatty acyl contents, TAG contents and the proportions of TAG in total lipids of *C. reinhardtii* **(A,B)** and *P. tricornutum* **(C,D)** following nitrogen starvation. The average values of duplicate culture replicates are shown. A total of 30 time points are shown for *C. reinhardtii* **(A)** and 21 time points for *P. tricornutum* **(B)**.

**FIGURE S2 |** Variations of acyl compositions of the major lipids, MGDG **(A)**, DGDG **(B)**, DGTS **(C)**, and TAG **(D)**, in nitrogen-deprived *C. reinhardtii*. MGDG, DGDG and DGTS denote monogalactosyldiacylglycerol, digalactosyldiacylglycerol and diacylglycerol-*N, N, N*-trimethylhomoserine, respectively. The sampling time points are set as 0, 4, 6, 24, 48, and 72 h, respectively.

**FIGURE S3 |** Variations of the major lipids contents in nitrogen-deprived *C. reinhardtii*. MGDG, DGDG and DGTS denote monogalactosyldiacylglycerol, digalactosyldiacylglycerol and diacylglycerol-*N, N, N*-trimethylhomoserine, respectively. The sampling time points are set as 0, 4, 6, 24, 48, and 72 h, respectively.

**FIGURE S4 |** Correlation analyses of the relative abundances **(A)** and the absolute contents **(B)** of CFAs and TAG contents in *C. reinhardtii* under high salt stress conditions. The HS medium under high salt stress conditions refers to Li et al. (2010). The other parameters are the same as that of nitrogen-deprived *C. reinhadtii*. The sampling time points are set as 0, 24, 48, and 72 h, respectively.

**FIGURE S5 |** Correlation analyses of the relative abundances **(A)** and the absolute contents **(B)** of CFAs and TAG contents in *Nannochloropsis oceanica* under phosphate deprivation conditions. The culture conditions under phosphate deprivation for *N. oceanica* are the same as that under nitrogen stress for *P. tricornutum*. The sampling time points are set as 0, 7, and 14 days following phosphate starvation, and 7 days following phosphate recovery (after 14 days of phosphate starvation), respectively.
