# Supplementary material for: An Extended Approach to Quantify Triacylglycerol in Microalgae by Characteristic Fatty Acids
Source: Front Plant Sci. 2017 Nov 13;8:1949. doi: 10.3389/fpls.2017.01949 (PMC5693890; doi:10.3389/fpls.2017.01949)
Supplement: Supplementary file 1 [file Image_1.PDF]

## Supplemental Material

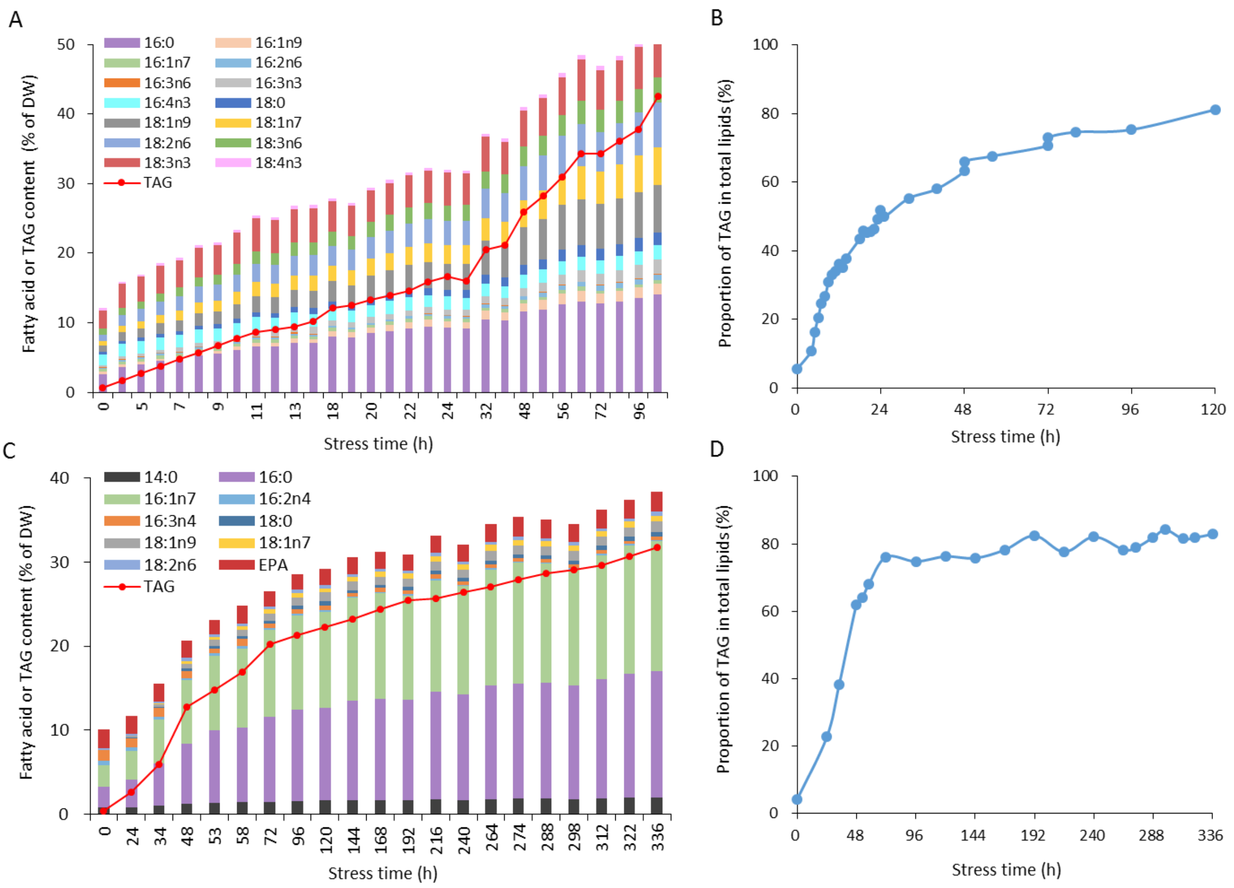


**Fig. S1.** Time course alterations of the fatty acyl profiles, TAG contents and the proportions of TAG in total lipids of *C. reinhardtii* (A, B) and *P. tricornutum* (C, D) following nitrogen starvation. The average values of duplicate culture replicates are shown. A total of 30 time points are shown for *C. reinhardtii* (A) and 21 time points for *P. tricornutum* (B).
