# Supplementary figures and images for: An Extended Approach to Quantify Triacylglycerol in Microalgae by Characteristic Fatty Acids
Source: Front Plant Sci. 2017 Nov 13;8:1949. doi: 10.3389/fpls.2017.01949 (PMC5693890; doi:10.3389/fpls.2017.01949)

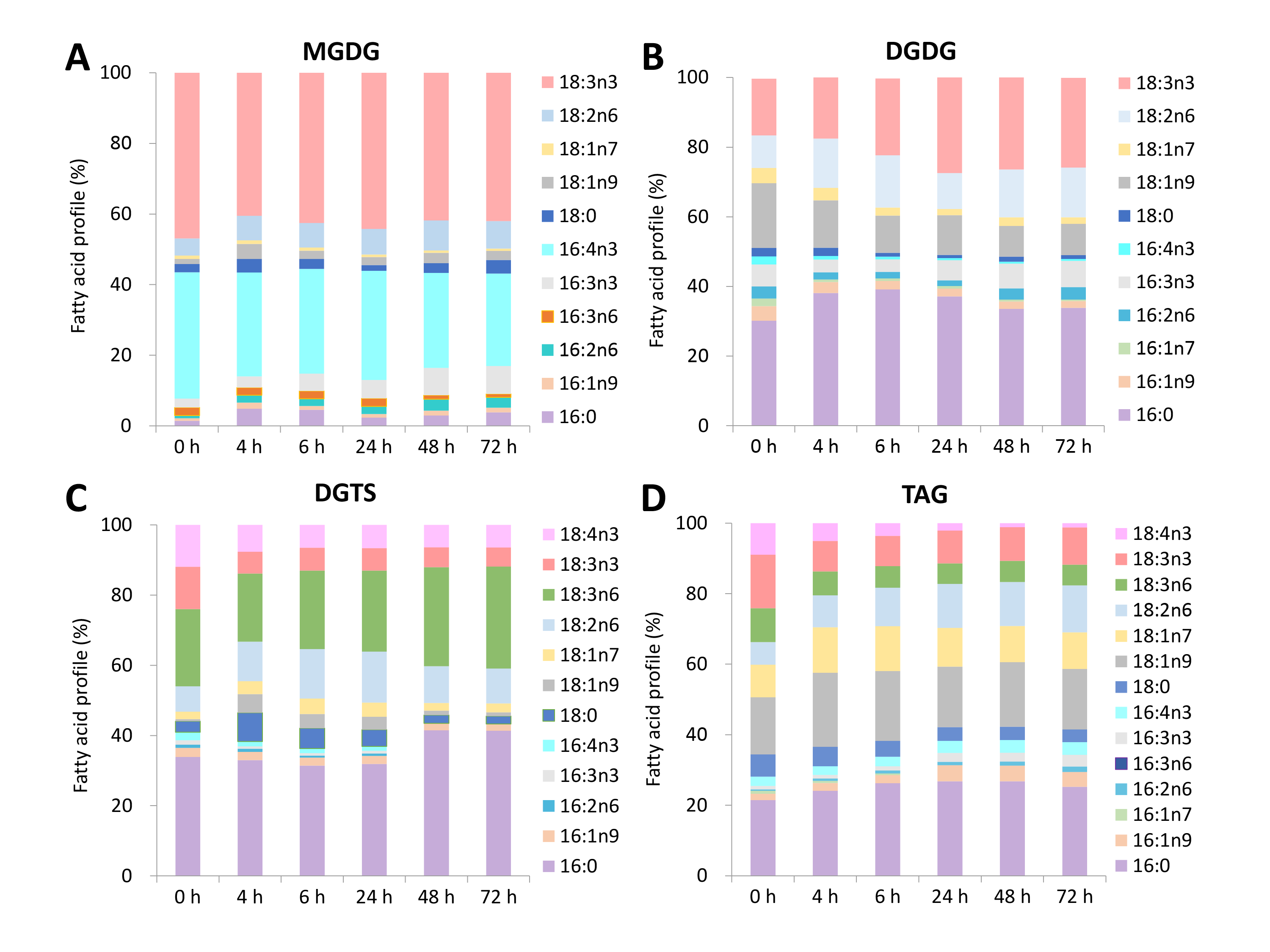

Supplement: Supplementary file 2 [file Image_2.TIF]

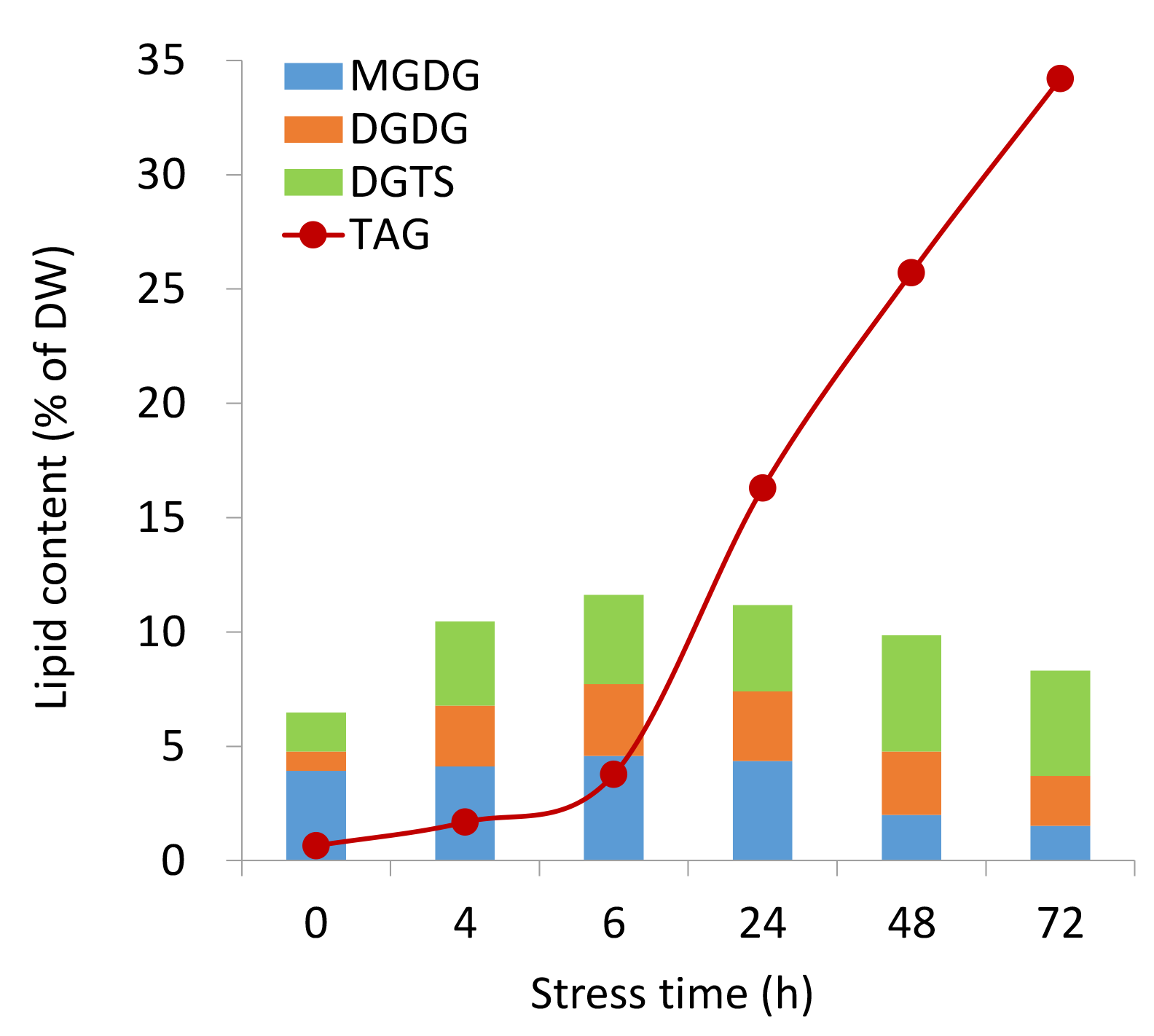

Supplement: Supplementary file 3 [file Image_3.TIF]

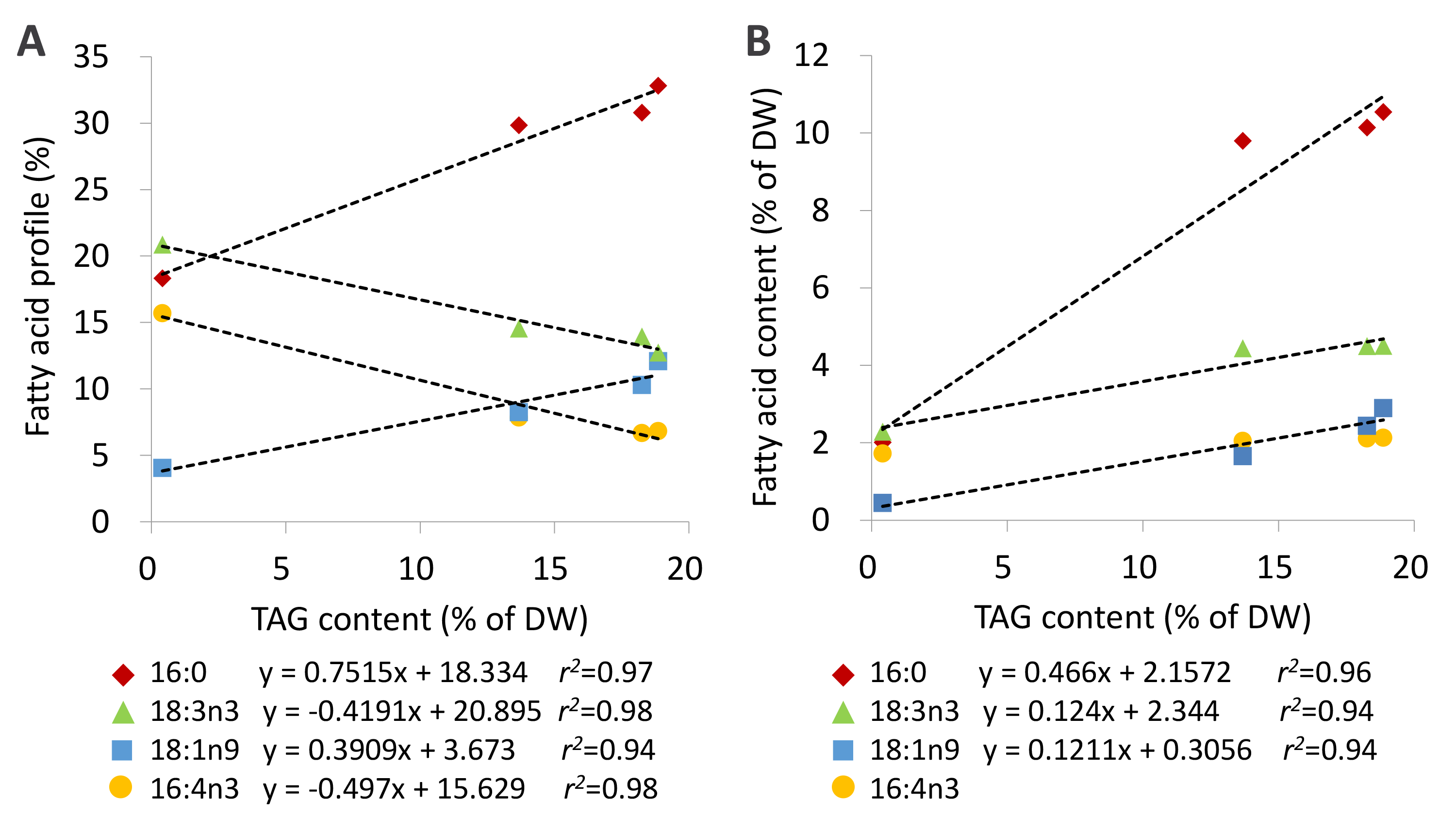

Supplement: Supplementary file 4 [file Image_4.TIF]

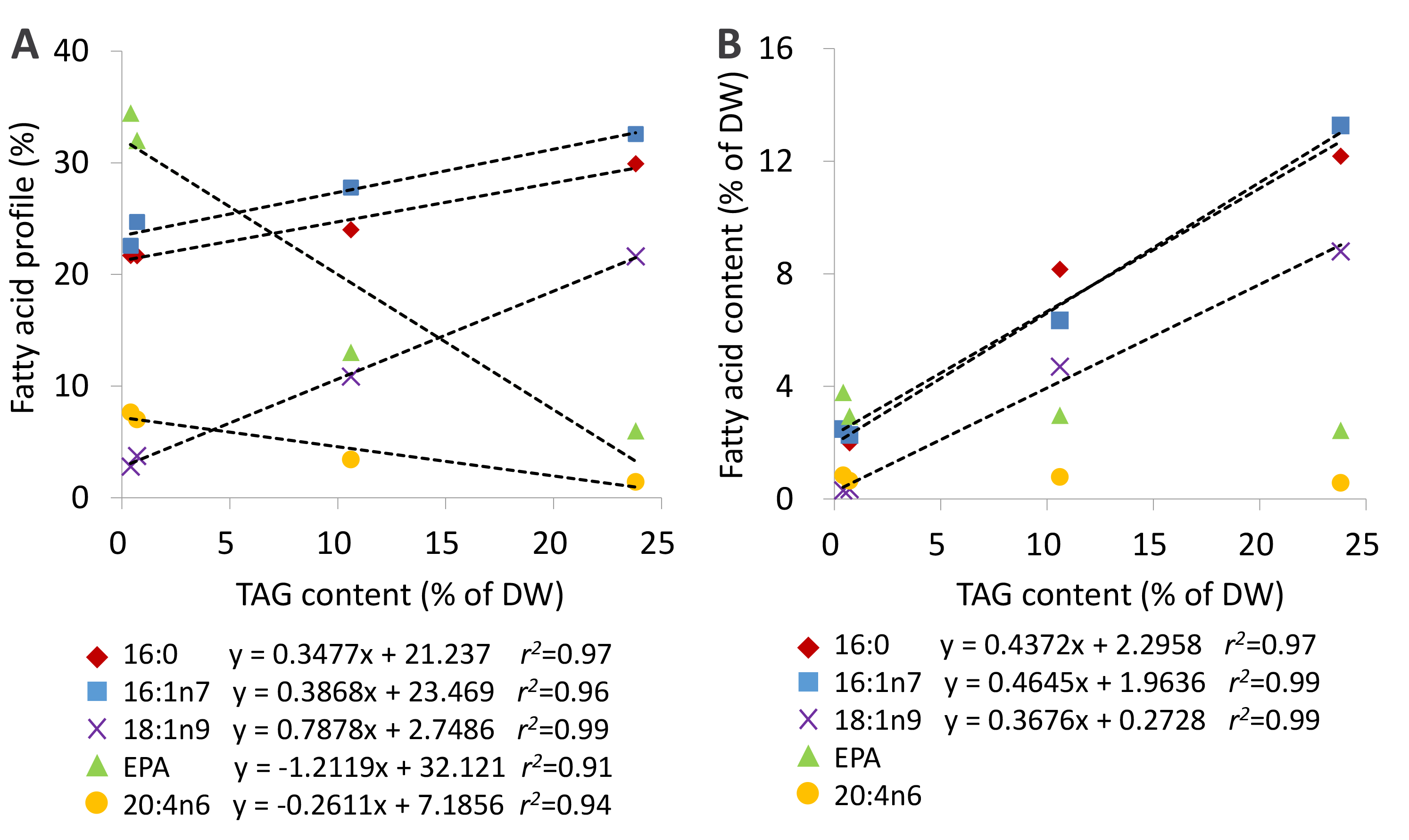

Supplement: Supplementary file 5 [file Image_5.TIF]
