# Supplementary material for: An Extended Approach to Quantify Triacylglycerol in Microalgae by Characteristic Fatty Acids
Source: Front Plant Sci. 2017 Nov 13;8:1949. doi: 10.3389/fpls.2017.01949 (PMC5693890; doi:10.3389/fpls.2017.01949)
Supplement: Supplementary file 6 [file Table_1.DOCX]

**Table S1** TAG fatty acyl profile of *C. reinhardtii* over the entire period of nitrogen starvation. Data are means±standard deviation of values for all time points.

| Fatty acid | % |
| --- | --- |
| 16:0 | 26±1 |
| 16:1n9 | 3±1 |
| 16:1n7 | 1±0 |
| 16:2n6 | 1±0 |
| 16:3n3 | 3±1 |
| 16:4n3 | 3±0 |
| 18:0 | 4±1 |
| 18:1n9 | 18±1 |
| 18:1n7 | 11±1 |
| 18:2n6 | 12±1 |
| 18:3n6 | 6±1 |
| 18:3n3 | 10±1 |
| 18:4n3 | 2±1 |
